# Pyroptosis of *Salmonella Typhimurium*-infected macrophages was suppressed and elimination of intracellular bacteria from macrophages was promoted by blocking QseC

**DOI:** 10.1038/srep37447

**Published:** 2016-11-17

**Authors:** Zhi Li, Qing Zheng, Xiaoyan Xue, Xin Shi, Ying Zhou, Fei Da, Di Qu, Zheng Hou, Xiaoxing Luo

**Affiliations:** 1 Department of Pharmacology, School of Pharmacy, Fourth Military Medical University, Xi’an 710032, P. R. China; 2 Center of Clinical Laboratory Medicine of PLA, Xijing Hospital, Fourth Military Medical University, Xi’an 710032, P. R. China

## Abstract

QseC is a membrane-bound histidine sensor kinase found in Gram-negative pathogens and is involved in the regulation of bacterial virulence. LED209, a QseC-specific inhibitor, significantly inhibits the virulence of several pathogens and partially protects infected mice from death by blocking QseC. However, the mechanism of its antibacterial effects remains unclear. In this experiment, a *Salmonella Typhimurium (S. Typhimurium*) and macrophage co-culture system was utilized to investigate possible mechanisms underlying the antimicrobial effects of the QseC inhibitor. QseC blockade inhibited the expression of QseC-dependent virulence genes, including *flhDC*, *sifA*, and *sopB*, in *S. Typhimurium*, leading to inhibition of swimming motility, invasion capacity, and replication capacity of the pathogens. Release of lactate dehydrogenase (LDH) from *S. Typhimurium*-infected macrophages was significantly inhibited by blocking QseC. Activated caspase-1 and IL-1β levels were suppressed, and intracellular bacterial count was reduced in infected macrophages. QseC blockade effectively reduced the virulence of *S. Typhimurium*, inhibited *S. Typhimurium*-induced pyroptosis of macrophages, and promoted elimination of intracellular bacteria from infected macrophages. Thus, the antibacterial effects of QseC inhibitor are mediated via enhancement of intracellular killing of *S. Typhimurium* in macrophages.

The rapid and global distribution of antimicrobial-resistant organisms is one of the leading causes of death in patients with severe infections and has become a significant challenge to humans^1^,^2^. Certain types of Gram-negative bacteria have become resistant to all available antibiotic drugs, contributing to increased morbidity and mortality, difficulty in controlling infectious illness, and escalating healthcare costs^3^,^4^. On the other hand, discovery and development of new antibiotics has rapidly declined, and the number of new antibiotics approved by the US Food and Drug Administration has decreased in recent years. Consequently, the arsenal of antibiotics is shrinking, weakening our ability to treat infectious diseases^5^,^6^. Therefore, developing new antibiotic agents with low probabilities of inducing resistance is of paramount importance.

Anti-quorum sensing is a promising theoretical strategy to prevent bacterial infections because this type of targeting may reduce resistance selection. Many Gram-negative bacteria carry QseC, a highly conserved membrane histidine sensor kinase, to identify environmental cues and regulate the expression of virulence factors. Upon sensing both host-derived adrenergic signals and the bacterial aromatic signal autoinducer-3, QseC autophosphorylates and subsequently phosphorylates a transcription factor, namely, QseB, which activates the transcription of key virulence genes^7^. Blockade of QseC by LED209, a selective inhibitor of QseC, significantly inhibits QseC-mediated activation of virulence-related gene expression and partially protects mice from death following infection with *Salmonella typhimurium* or *Francisella tularensis*^8^. However, LED209 only inhibits bacterial virulence and does not suppress *S. Typhimurium* growth. The fundamental mechanism underlying the *in vivo* protective effects of LED209 remains unclear.

The macrophage inflammasome, a critical component of the innate immune response, is a large multiprotein complex that recognizes invading pathogens in the cytosol and enables autocatalytic activation of caspase-1. Activation of this protein triggers maturation and release of the pro-inflammatory cytokines IL-1β and IL-18 as well as pyroptosis, a pro-inflammatory form of macrophage cell death^9^,^10^. Previous studies reported that the macrophage NLRC4 inflammasome can detect *S. Typhimurium* flagellin. Moreover, caspase-1-induced pyroptosis of macrophages is an innate immune response to fight intracellular bacteria^11^. However, excessive cell pyroptosis can cause immunological diseases and septic shock in the host^12^,^13^.

We hypothesized that QseC inhibitors may suppress inflammasome over-activation and macrophage pyroptosis by inhibiting bacterial virulence release and enhancing the clearance of *S. Typhimurium*. In this experiment, we investigated the possible antimicrobial mechanisms of Br-LED209, a LED209-derived QseC inhibitor, with a co-culture system of *S. Typhimurium* and macrophages. Br-LED209 effectively blocked the QseC of *S. Typhimurium* and suppressed the expression of its virulence genes. After that, QseC blockade inhibited *S. Typhimurium*-induced pyroptosis of macrophages and promoted the elimination of intracellular bacteria, which were possibly mediated by inhibiting excessive activation of inflammasomes in the infected macrophages.

## Materials and Methods

### Bacterial strains

*S. Typhimurium* (XJ76218), enterohemorrhagic *Escherichia coli* O157:H7 (EHEC) (XJ76330), and *Shigella flexneri* (XJ76116) were obtained from Xijing Affiliated Hospital of the Fourth Military Medical University (Xi’an, China). All of these strains were isolated from feces of patients.

### Synthesis and characterization of Br-LED209

Ara-acetylaminobenzene sulfonyl chloride and para-bromoaniline were used as raw materials to obtain 4-amino-N-(4-bromophenyl)benzenesulfonamide through acylation and deacylation. The resulting product and phenyl isothiocyanate were then used to yield N-(4-bromophenyl)-4-(3-phenyl thiourea)benzenesulfonamide, named Br-LED209 in short. The synthesis route and chemical structure of Br-LED209 is shown in Figure S1. The chemical structure of Br-LED209 was confirmed by mass spectrometry (MS), ^1^H NMR, and ^13^C NMR (data not shown). Br-LED209 is a light yellow powder with melting point between 116 °C and 120 °C. The purity of Br-LED209 is higher than 95%. Br-LED209 was dissolved in DMSO and the final concentration of DMSO in all of the *in vitro* experiments was 1% (v/v). DMSO at the same concentration was used as control in all the experiments.

### Construction of the *qseC* mutant strain

An isogenic *S. Typhimurium qseC* mutant was constructed using λ red mutagenesis as described previously^8^. Briefly, a *qseC* PCR product was generated using primers showed in Table 1 and pKD3 as template, and gel-purified. The PCR product was electroporated into *S. Typhimurium* containing pKD46, which were then incubated at 37 °C for 1 hour, and plated on media containing 30 μg/ml chloramphenicol overnight at 37 °C. The resultant colonies were patched for chloramphenicol resistance and ampicillin sensitivity, and PCR verified for the absence of the gene. Plasmid pCP20, which encodes a resolvase, was electroporated into the mutant strain, and the resulting colonies were patched for chloramphenicol sensitivity. The chloramphenicol cassette was resolved from the mutant and a non-polar isogenic *qseC* mutant was created.

### Bacterial susceptibility and growth assay

To analyze bacterial growth, one isolated single colony was inoculated into 2 ml of Luria–Bertani (LB) broth and cultured at 37 °C with shaking overnight. The overnight culture was diluted to 1:100 in 4 ml of fresh LB broth and then cultured at 37 °C. Br-LED209 was added to bacterial cultures to obtain final concentrations of 10, 50, and 200 μM. Bacterial cultures were collected for cell growth analysis by measuring OD_600_ absorbance every 1 h, and growth curves were generated according to the values of OD_600_ absorbance. The minimum inhibitory concentrations (MICs) were determined by a microdilution assay with broth microdilution guidelines published by the Clinical and Laboratory Standards Institute. Levofloxacin (LEV) was used as the positive antibiotic control.

### Ethics statement

The experimental and animal care procedures were approved by the Animal Care and Use Committee of the Fourth Military Medical University. All procedures were carried out in strict accordance with the approved guidelines.

### Protective effects of Br-LED209 on *S. Typhimurium*-infected mice

Eight- to ten-week-old male BALB/c mice weighing 18–22 g were used in this study. Infection was induced by intraperitoneal administration of 1.0 × 10^8^ colony-forming units (CFUs) of wild-type (WT) *S. Typhimurium* or the *qseC* mutant in 0.4 ml of LB broth. The mice were treated orally with 20 mg/kg Br-LED209 three hours before and after infection. Livers and spleens were harvested at 8, 16 and 24 hours after infection. The samples were homogenized, and the supernatants were plated on agar plates for evaluating bacterial burden in the infected organs. The morphologies of livers and spleens of infected mice were examined by observing hematoxylin and eosin-stained sections of the respective tissues.

### Quantitative real-time RT-PCR

To evaluate the QseC blocking efficiency of Br-LED209, the expression of virulence genes in WT *S. Typhimurium* and the *qseC* mutant was detected by quantitative real-time PCR. In brief, RNA was extracted from an overnight culture grown aerobically in LB medium in the absence or presence of 200 μM Br-LED209 using a bacterial RNA isolation kit (Tiangen) according to the manufacturer’s protocol. The primers used in the real-time assays are listed in Table 2. Real-time RT-PCR was performed with SYBR Premix Ex TaqII (Takara) following the manufacturer’s instruction. The *rpoA* (RNA polymerase subunit A) gene was used as the endogenous control. Data was collected using the Bio-Rad CFX Manager 2.1 software. Data were normalized to levels of *rpoA* and analyzed by the comparative critical threshold (C_T_) method. Expression levels in different groups were compared by the relative quantification method. Real-time data are expressed below as fold changes compared with the WT group in the absence of Br-LED209.

### Motility assay

To evaluate the effect of Br-LED209 on the flagella motility of *S. Typhimurium*, *S. Typhimurium* (WT and *qseC* mutant) was cultured in LB medium for 12 h. Subsequently, 1 μl aliquot of the culture was spotted onto 0.3% agar plates with or without 200 μM Br-LED209, and halo sizes were measured at 6, 12 and 18 hours after incubation at 37 °C^14^.

### HeLa cell invasion and intracellular bacteria replication assay

HeLa cells were plated in 96-well culture dishes at a concentration of 2 × 10^4^ cells/well and infected with WT *S. Typhimurium* or the *qseC* mutant at a multiplicity of infection (MOI) of 50:1 for 1 h. The cells were then treated with 100 μg/ml gentamicin for 1 h to kill extracellular bacteria. The cell culture medium was replaced with 10 μg/ml gentamicin for the remainder of the experiment. The cells were then lysed with 1% Triton X-100 at the indicated time points, and the lysates were diluted and plated on agar plates to determine the number of CFUs^14^. Fold increase was calculated as the ratio of intracellular bacteria at 6 and 12 hours to that at 1 hour.

### Macrophage release lactate dehydrogenase (LDH) assay

Male BALB/c mice weighing 18–22 g were sacrificed by cervical dislocation. Resident peritoneal macrophages were harvested as described previously with slight modification^15^. In brief, the peritoneal cavity was lavaged with 5 ml of ice-cold sterile PBS. The buffer containing resident peritoneal cells was slowly withdrawn. Cells were collected by centrifugation (1000 × g for 5 min) and plated in a culture flask containing Dulbecco’s minimal essential medium (Hyclone) supplemented with 10% fetal bovine serum, 100 U/ml penicillin, and 100 μg/ml streptomycin. The peritoneal macrophages were allowed to adhere for 3 h (37 °C, 5% CO_2_) and then washed with PBS to remove unattached cells.

The macrophages were plated in 96-well culture dishes (Falcon, Franklin Lakes, NJ, USA) at a concentration of 5 × 10^4^ cells/well. Experiments were performed as described previously with slight modifications^16^,^17^,^18^. Briefly, *S. Typhimurium* were grown overnight in LB at 37 °C. The overnight culture was subsequently diluted at 1:50 into fresh LB and grown for 3–4 h at 37 °C before infection. Macrophages were infected with WT *S. Typhimurium* or the *qseC* mutant diluted in fresh DEME at MOI of 25:1. Images of macrophages were taken under a light microscope at 1 h after infection. LDH released in the supernatant was detected using a cytotoxicity detection kit (Roche). Data on detected LDH were used to calculate the pyroptotic rate of infected macrophages based on the following equation: [(experimental release − spontaneous release)/(maximum release − spontaneous release)] × 100, where spontaneous release is from the cytoplasm of uninfected macrophages, and maximum release is that obtained by lysis of macrophages with a solution of 0.1% Triton X-100.

### Fluorescence microscopy

For the fluorescence microscope assay, macrophages seeded on a glass slide were infected with WT *S. Typhimurium* or the *qseC* mutant at MOI of 25:1 for 1 h and washed twice with PBS. The macrophages were stained with DAPI and the membrane impermeant dye propidium iodide (PI). The percentage of PI-positive cells was determined by counting cell numbers in four random visual fields.

### Western blot

The caspase-1 p10 subunit and processed interleukin (IL)-1β released into the culture supernatant from macrophages were collected and precipitated with 10% TCA (vol/vol) for 1 h on ice. Precipitated proteins were pelleted at 20,000× g for 15 min at 4 °C, washed with ice-cold acetone, air-dried, resuspended in SDS-PAGE sample buffer, and heated to 95 °C for 10 min. Proteins were loaded and separated on 15% SDS-polyacrylamide gel and then transferred to polyvinylidene difluoride membranes (Millipore Corporation, Billerica, MA, USA). Western blots were performed with rabbit anti-mouse caspase-1 antibody (Epitomics, EPR4321) diluted to 1:5000 and goat anti-mouse IL-1β antibody (R&D Systems, AF-401-NA) diluted to 1:2000. Cell lysates were probed with anti-β-actin antibodies (Sigma, Cat. No. A2066) diluted to 1:5000.

### Macrophage infection assay

Macrophage infection experiments were performed as described previously with some modifications^19^,^20^,^21^. In brief, macrophages were plated in 96-well culture dishes at a concentration of 5 × 10^4^ cells/well. *S. Typhimurium* were cultured at 37 °C with shaking overnight and were opsonized in DMEM and 10% normal mouse serum for 20 min. The macrophages were infected with opsonized WT *S. Typhimurium* or the *qseC* mutant at MOI of 25:1 for 30 min. Then the cells were washed with PBS and treated with 100 μg/ml gentamicin for 1 h to kill extracellular bacteria. The cell culture medium was replaced with 10 μg/ml gentamicin for the remainder of the experiment. To count the number of intracellular bacteria, macrophages were washed three times with PBS and lysed with 1% Triton X-100 for 10 min. Bacteria were diluted and plated on LB medium plates to determine the number of CFU at indicated time points.

### Statistical analysis

Data are shown as mean ± SD. One-way ANOVA and two-way ANOVA were used to evaluate statistical significance. A probability value of *P* < 0.05 was considered statistically significant.

## Results

### Bacterial growth was not inhibited by Br-LED209 *in vitro*

**Figure 1:**
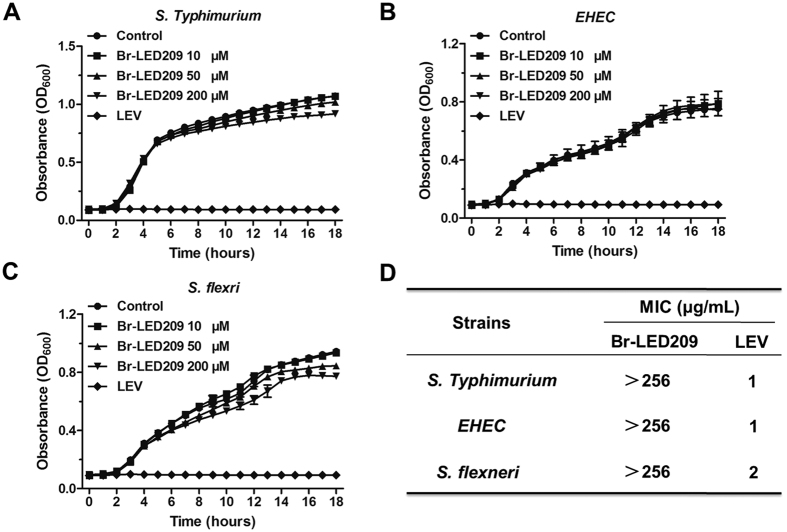
Br-LED209 has no influence on bacterial growth *in vitro*. Growth curves of *Salmonella Typhimurium* (**A**) *Enterohemorrhagic Escherichia coli* (EHEC) (**B**) *Shigella flexneri* (**C**) were drawn every hour at OD_600_ absorbance in the absence or presence of Br-LED209 at different concentrations. The antibiotic levofloxacin (LEV) was used as positive control. (**D**) Minimal inhibitory concentration (MIC) of Br-LED209.

To assess whether Br-LED209 influences the growth of bacteria *in vitro*, *S. Typhimurium, S. flexneri*, and EHEC were cultured in LB medium in the absence or presence of Br-LED209 at different concentrations. The growth curve demonstrated that Br-LED209 did not inhibit growth of the three strains *in vitro* at concentrations up to 200 μM. Meanwhile, levofloxacin, which was used as a positive control, completely inhibited the growth of all three tested strains at two fold dilutions of MIC values used on each strain (Fig. 1A–C). The results of MIC assay showed that Br-LED209 did not have significant bactericidal effects at a concentration of 256 μg/ml *in vitro* (Fig. 1D).

### *S. Typhimurium*-infected mice were protected *in vivo* by blocking QseC

**Figure 2:**
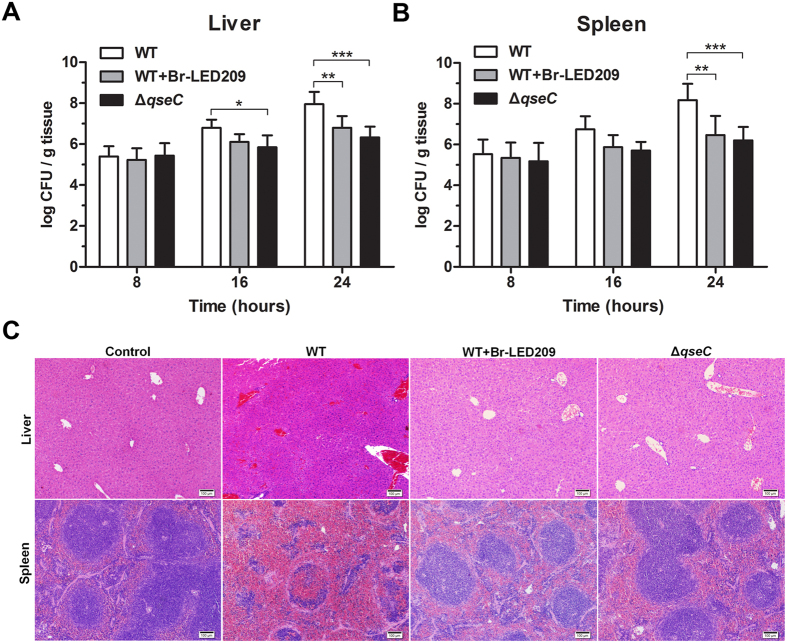
Bacterial burden and pathological damages in organs of infected mice were reduced by blocking QseC. (**A**,**B**) BALB/c mice were infected by intraperitoneal administration of 1.0 × 10^8^ CFUs of WT *S. Typhimurium* or the *qseC* mutant. The mice were treated twice orally with Br-LED209 (20 mg/kg) at 3 h before and after infection. Livers and spleens were harvested at 8, 16 and 24 hours after infection, homogenized and then plated on agar plates for bacterial counts. (*P < 0.05, **P < 0.01, ***P < 0.001 vs. WT in two-way ANOVA, n = 5) (**C**) Morphological characteristics of liver and spleen in BALB/c mice were compared among groups infected with WT *S. Typhimurium*, the *qseC* mutant or Br-LED209. Scale bars: 100 μm.

To evaluate the protective effects of Br-LED209 *in vivo*, BALB/c mice were infected by intraperitoneal administration of WT *S. Typhimurium* or the *qseC* mutant at 1.0 × 10^8^ CFUs. The mice were treated twice orally with Br-LED209 (20 mg/kg) at 3 h before and after infection. Livers and spleens were harvested at 8, 16 and 24 hours after infection, homogenized and then plated on agar plates for bacterial counts. The results showed that bacterial numbers in the liver and spleen were dramatically lower in Br-LED209-treated mice and *qseC* mutant-infected mice than the corresponding values in WT-infected mice (Fig. 2A,B).

Livers and spleens were harvested at 24 hours after infection and stained with hematoxylin and eosin. The morphological structure of liver tissue samples exhibited obvious hepatocyte edema and vacuolar degeneration in the WT-infected group. Besides, the WT-infected group showed significant congestion in hepatic sinusoid and central vein. No obvious hepatocyte edema, vacuolar degeneration and congestion were observed in the Br-LED209-treated and the *qseC* mutant-infected groups (Fig. 2C). In spleen tissue, splenic corpuscles demolished and disappeared, red pulp widened and white pulp atrophied in the WT-infected group. Meanwhile, obvious congestion was observed in the red pulp and white pulp area of the WT-infected group. However, hyperemia of red pulp and white pulp was drastically reduced in the Br-LED209-treated group and the *qseC* mutant-infected group (Fig. 2C).

### Swimming motility of *S. Typhimurium* was inhibited by blocking QseC

**Figure 3:**
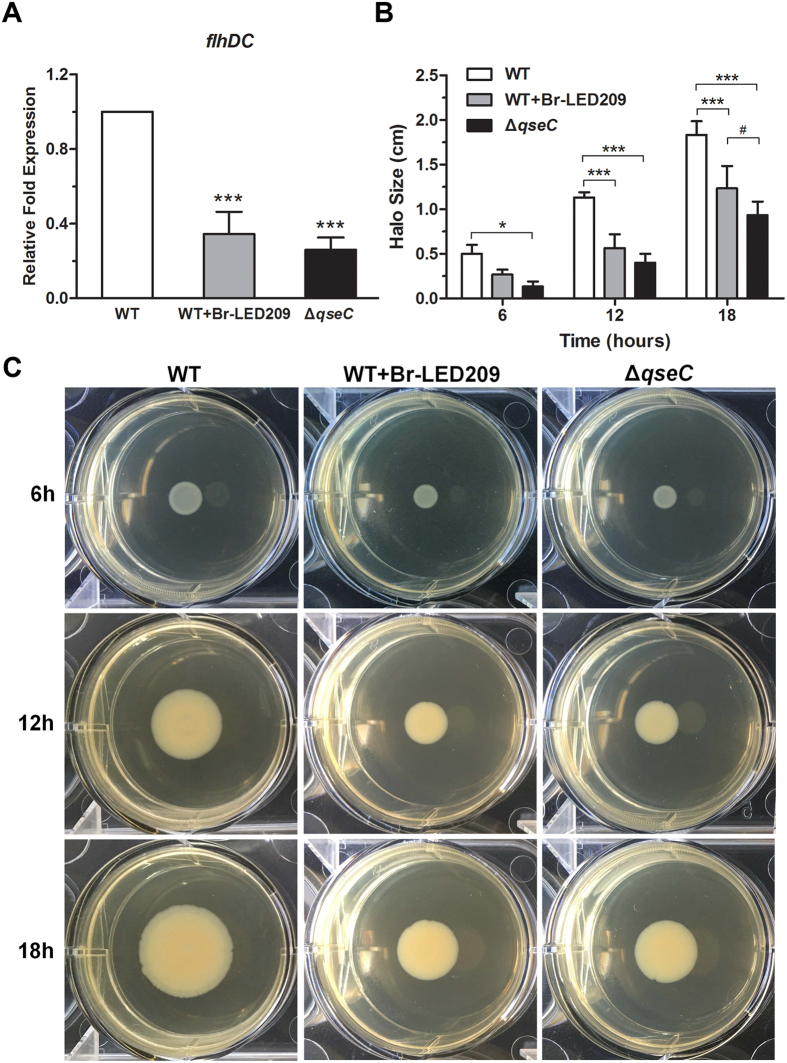
The swimming motility of *S. Typhimurium* was inhibited by blocking QseC. (**A**) Expression of the mobility-related gene *flhDC* was evaluated. RNA was extracted from an overnight culture grown aerobically in LB medium in the absence or presence of Br-LED209. Real-time reverse transcription (RT)-PCR was performed. The *rpoA* gene was used as the endogenous control. Data were normalized to levels of *rpoA* and were calculated as fold changes compared to the WT group. (***P < 0.001 vs. WT in one-way ANOVA, n = 3) (**B**,**C**) Swimming motility assay. About 1 μl of WT bacteria or the *qseC* mutant was spotted onto agar plates with or without 200 μM Br-LED209, and halo sizes were measured at 6, 12 and 18 hours after incubation at 37 °C. (*P < 0.05, ***P < 0.001 vs. WT; ^#^*P* < 0.05 vs. WT+Br-LED209 in two-way ANOVA, n = 3).

Flagella-mediated motility is fundamental to *S. Typhimurium* pathogenesis. For this reason, expression of the *flhDC* gene, which encodes the master regulator of flagellum biosynthesis, was evaluated by quantitative real-time PCR. Data were normalized to levels of *rpoA* and were calculated as fold changes compared to the WT group. The results showed that *flhDC* expression in *S. Typhimurium* was dramatically decreased in the presence of Br-LED209 at 200 μM and also the *qseC* mutant compared with the WT strain (Fig. 3A). Next, a motility assay was performed to examine whether swimming motility of *S. Typhimurium* was affected. The diameter of motility halos was significantly reduced in the presence of Br-LED209 at 200 μM and also in the *qseC* mutant compared with the WT strain (Fig. 3B,C). These motility experiments were performed in triplicate, and the halo diameters reflect the average of these experiments. These results suggested that motility inhibition of *S. Typhimurium* was related to QseC blockade.

### The invasion and replication capacities of *S. Typhimurium* were inhibited by blocking QseC

**Figure 4:**
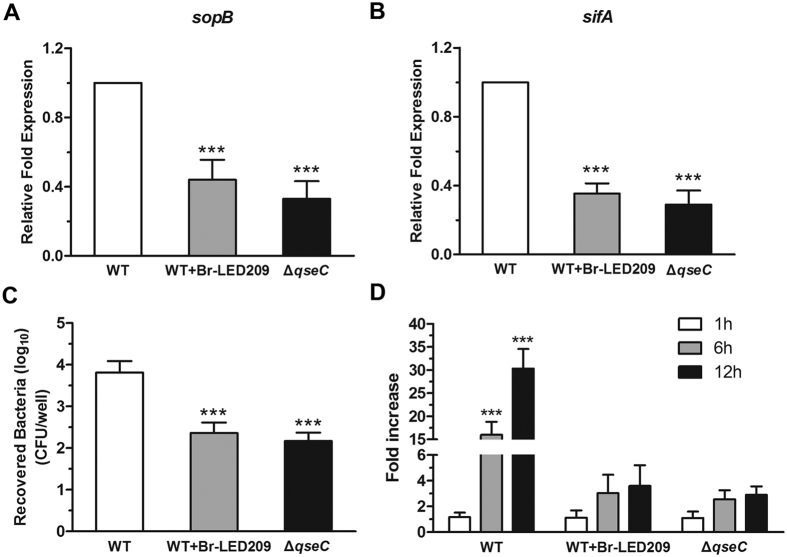
The invasion and replication capacities of *S. Typhimurium* were inhibited by blocking QseC. (**A**,**B**) Expression of the invasion- and replication-related genes *sopB* and *sifA* was evaluated in *S. Typhimurium*. RNA was extracted from an overnight culture grown aerobically in LB medium in the absence or presence of Br-LED209, and RT-PCR was performed. The *rpoA* gene was used as the endogenous control. Data were normalized to levels of *rpoA* and were calculated as fold changes compared to the WT group. (***P < 0.001 vs. WT in one-way ANOVA, n = 3) (**C**) HeLa cells were infected with *S. Typhimurium* for 1 h. The cells were treated with 100 μg/ml gentamicin for 1 h to kill extracellular bacteria. Then the cells were lysed and bacteria were diluted and plated on LB medium plates to determine the number of CFU. (***P < 0.001 vs. WT in one-way ANOVA, n = 3) (**D**) After treatment with 100 μg/ml gentamicin to kill extracellular bacteria, the cell culture medium was replaced with 10 μg/ml gentamicin for the remainder of the experiment. Cells were lysed at the indicated time points and intracellular bacteria were counted. Fold-increase was calculated as the ratio of intracellular bacterial count at 6 and 12 hours to that 1 hour. (***P < 0.001 vs. WT in two-way ANOVA, n = 3).

To evaluate the invasion and replication capacity of *S. Typhimurium*, the expression of invasion- and replication-associated genes *sopB* and *sifA* in *S. Typhimurium* were measured by quantitative real-time PCR. Data were normalized to levels of *rpoA* and calculated as fold changes compared with the WT group. The results showed that QseC blockade inhibited the expression of *sopB* and *sifA* in *S. Typhimurium* (Fig. 4A,B). To further investigate the down-regulation of invasion-associated genes in response to QseC blockade, the invasion capacity and replication capacity of *S. Typhimurium* on epithelial cells were investigated. HeLa cells were infected with WT *S. Typhimurium* or the *qseC* mutant at the MOI of 50:1 for 1, 6, or 12 h. The cells were lysed with 1% Triton X-100, and the lysates were diluted and plated on agar plates to determine the CFU. Fold-increase was calculated as a ratio of the intracellular bacteria count at 6 and 12 hours to that at 1 hour. The results demonstrated that QseC blockade significantly decreased the numbers of intracellular bacteria at each time point (Fig. 4C,D), suggesting that the invasion and replication capacities of *S. Typhimurium* were inhibited by blocking QseC.

### Inflammasome activation and pyroptosis of infected macrophages was inhibited by blocking QseC

**Figure 5:**
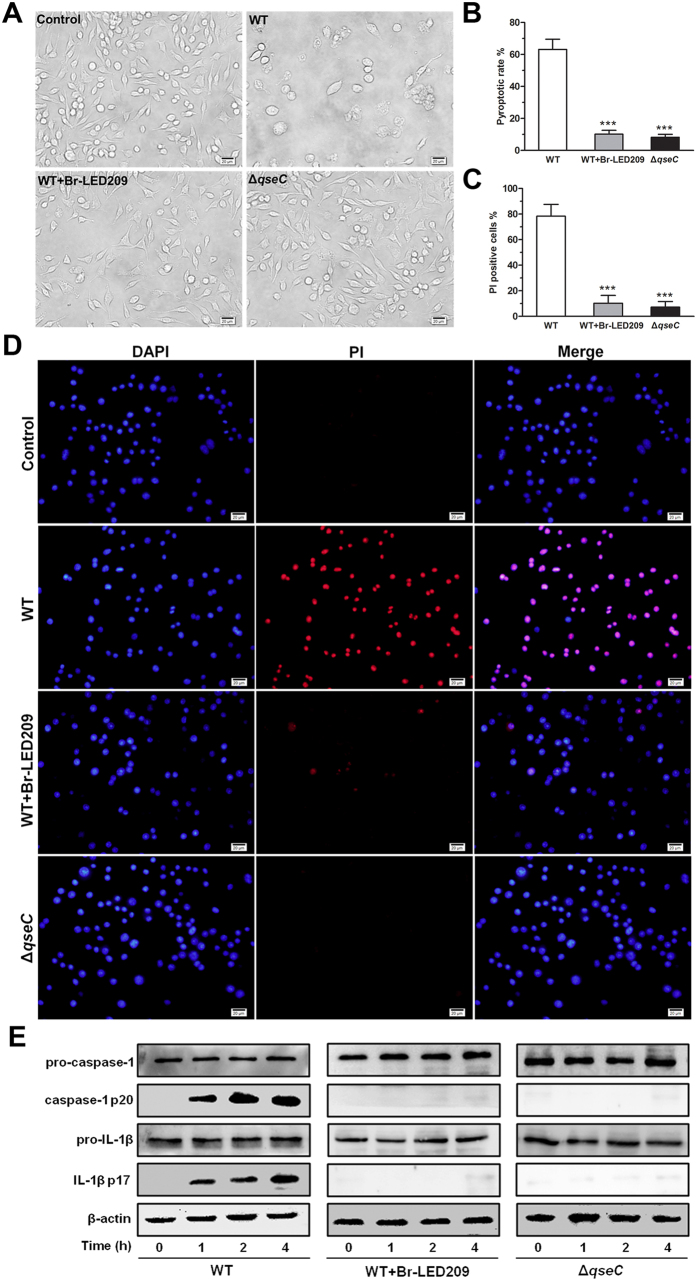
Inflammasome activation and pyroptosis of infected macrophages was inhibited by blocking QseC. (**A**) *S. Typhimurium* were grown overnight in LB and subsequently diluted at 1:50 into fresh LB and grown for 3-4 h at 37 °C. Macrophages were infected with WT *S. Typhimurium* or the *qseC* mutant diluted in fresh DEME at MOI of 25:1. Morphological characteristics of macrophages after *S. Typhimurium* infection. Light microscope images were taken at 1 hour post-infection. Scale bars: 20 μm. (**B**) LDH released from *S. Typhimurium*-infected macrophages was measured. The pyroptotic rate of macrophages was calculated based on the equation in methods. (***P < 0.001 vs. WT in one-way ANOVA, n = 3) (**C**,**D**) Membrane permeability of macrophages after *S. Typhimurium* infection. Macrophages were stained with DAPI and the membrane impermeant dye propidium iodide (PI). Scale bars: 20 μm. The percentage of PI-positive cells was determined by counting cell numbers in four random visual fields. (***P < 0.001 vs. WT in one-way ANOVA) (**E**) Macrophages were infected with *S. Typhimurium*. Activated caspase-1 and IL-1β were precipitated from macrophage supernatants and detected by Western blot.

Br-LED209 did not kill *S. Typhimurium in vitro* (Fig. 1), but eliminated them from the internal organs of infected mice (Fig. 2). We hypothesized that the clearance of bacteria from infected mice by Br-LED209 might be mediated by antimicrobial innate immune response. Macrophages play crucial role in controlling *S. Typhimurium* infection. Therefore, we investigated whether the function of macrophages was affected by *S. Typhimurium* infection. Overnight culture of *S. Typhimurium* were diluted at 1:50 into fresh LB and grown for 3–4 h at 37 °C before the infection. Macrophages were infected with WT *S. Typhimurium* or the *qseC* mutant at MOI of 25:1. Images of macrophages were taken under a light microscope at 1 h after infection. The images showed that a high percentage of macrophages infected with *S. Typhimurium* were swollen, and many of the infected cells had died at 1 h after infection. In the Br-LED209-treated group and the *qseC* mutant-infected group, macrophages were protected and no significant cell death was observed (Fig. 5A). LDH released in the supernatant was detected 1 h after infection using a cytotoxicity detection kit. Results showed that the amount of released LDH decreased significantly after QseC blockade (Fig. 5B). To further confirm these results, we stained the infected macrophages with DAPI and PI and calculated the percentage of PI-positive cells by counting cell numbers in four random visual fields. We detected strong PI signals in *S. Typhimurium*-infected macrophages. QseC blockade significantly reduced the number of PI-positive cells (Fig. 5C,D). The inflammasome can recognize invading pathogens, activate caspase-1 and subsequently trigger inflammatory cell death known as pyroptosis. To further confirm the manner of death of infected macrophages, the expression of activated caspase-1 and IL-1β were detected by Western blot. Data showed that QseC blockade significantly suppressed the production of the caspase-1 p10 and IL-1β p17 forms of macrophages (Fig. 5E), which indicated that inflammasome activation had been significantly inhibited and macrophage pyroptosis had been prevented.

### Elimination of intracellular bacteria from macrophages was enhanced by blocking QseC

**Figure 6:**
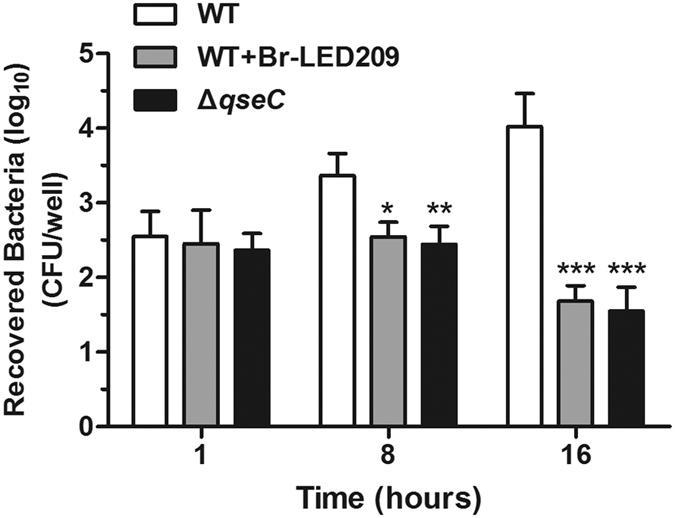
Elimination of intracellular bacteria from macrophages was enhanced by blocking QseC. *S. Typhimurium* were cultured at 37 °C with shaking overnight and opsonized in DMEM and 10% normal mouse serum for 20 min. Macrophages were infected with opsonized WT *S. Typhimurium* or the *qseC* mutant at MOI of 25:1 for 30 min. The cells were washed with PBS and treated with 100 μg/ml gentamicin for 1 h to kill extracellular bacteria. Then, cell culture medium was replaced with 10 μg/ml gentamicin for the remainder of the experiment. To count the number of intracellular bacteria, macrophages were lysed with 1% Triton X-100. Bacteria were diluted and plated on LB medium plates to calculate CFU at the indicated time points. (*P < 0.05, **P < 0.01, ***P < 0.001 vs. WT in two-way ANOVA, n = 3).

Next, the capacity of the macrophages to kill bacteria was evaluated. Since inflammasome-activating *S. Typhimurium* induced rapid macrophage pyroptosis within 1 hour of infection (Fig. 5), intracellular bacterial burden could not be measured under this condition. Thus, another condition was used to study macrophage-killing capacity according to many previously published studies^19^,^20^,^21^. *S. Typhimurium* were cultured overnight and opsonized in DMEM containing 10% normal mouse serum for 20 min before infection. The numbers of intracellular vital bacteria were counted at 1, 8, and 16 hours after infection. The data showed no difference in the number of recovered bacteria among the different treatment groups at one hour after infection. However, the number of bacteria recovered from macrophages significantly decreased after Br-LED209 treatment or infection with *qseC* mutant bacteria at 8 and 16 hours after infection (Fig. 6). The results indicated that QseC blockade could promote the clearance of intracellular *Salmonella* by inhibiting intracellular replication of bacteria and increasing the killing capacity of macrophages.

## Discussion

QseC is a promising target for developing broad-spectrum antimicrobials because of its broad distribution and high degree of conservation among different bacterial species^22^. LED209 is a potent QseC inhibitor that blocks the expression of virulence genes and suppresses the pathogenicity of Gram-negative bacteria^8^,^22^. Thus, LED209 is a promising antimicrobial agent. However, LED209 only exerts protective effects against *S. Typhimurium* infection in a mouse model and does not affect bacterial growth *in vitro*. The mechanism underlying this phenomenon remains unclear.

In this study, Br-LED209, which is a LED209 derivative, was synthesized to explore the mechanism of its protective effects *in vivo*. The efficacy of Br-LED209 at inhibiting QseC was then evaluated *in vitro* and *in vivo*. Results showed that Br-LED209 did not kill *S. Typhimurium in vitro* (Fig. 1). However, bacterial burden in the liver and spleen of infected mice was reduced significantly and pathological damage of these infected organs was alleviated after Br-ED209 treatment *in vivo* (Fig. 2). These results indicated that Br-LED209 retained its ability to block QseC.

QseC regulates multiple virulence factors involved in the pathogenesis of *S. Typhimurium*. As such, downstream genes regulated by QseC would be affected if the protective effects of Br-LED209 were mediated by blocking QseC. The *flhDC* gene encodes the master regulator of flagellum biosynthesis in *S. Typhimurium*, and regulates bacterial motility and facilitates bacterial invasion^23^. The *sopB* gene encoding SopB participates in the invasion of nonphagocytic cells, early maturation of *Salmonella*-containing vacuole (SCV), regulation of SCV trafficking, and inhibition of SCV–lysosome fusion^24^,^25^. The *sifA* gene is required for SCV membrane integrity and SCV maintenance. This gene permits the survival and replication of *S. Typhimurium* in macrophages and inhibits lysosome function^20^,^26^. All of these three genes are tightly regulated by QseC and are particularly important in *S. Typhimurium* pathogenesis. Therefore, the expression and virulence of these genes were investigated. Results indicated that QseC blockade significantly inhibited the expression of QseC-regulated virulence genes including *flhDC*, *sifA*, and *sopB* in *S. Typhimurium*. Moreover, QseC blockade suppressed flagellar motility as well as the invasion and replication capacities of *S. Typhimurium* in epithelial cells (Figs 3 and 4).

Macrophages are key innate immune cells that play a critical role in controlling bacterial infection. Inflammasome signaling of macrophages and caspase-1-induced pyroptotic cell death are innate immune effector mechanisms against many bacterial species^11^,^27^,^28^,^29^,^30^. Pyroptosis removes the replication niche of intracellular bacteria and promotes bacterial clearance through the NADPH oxidase system of recruited neutrophils^11^. Previous studies have reported that caspase-1 activation clears pathogens such as *Salmonella*^28^, *Shigella*^29^, and *Legionella*^30^, and controls infection.

However, systemic or excessive activation of inflammasome signaling and pyroptosis *in vivo* may be deleterious to bacterial clearance^12^,^13^. For example, *Shigella* infection activated NLR inflammasomes, promoted macrophage cell death, and secured its own dissemination^31^. In a murine model of acute pneumonia, pyroptosis of alveolar macrophage by inflammasome signaling induction impaired *Pseudomonas aeruginosa* clearance and increased mortality. Conversely, restricting inflammasome activation enhanced bacterial clearance and decreased pathology^32^. NLRC4 inflammasome-induced pyroptotic signaling have been reported to cause enhanced vascular permeability, septic shock, and rapid death in mice after the cytosolic delivery of bacterial flagellin^33^. Ayres *et al.* found that inflammasome activation in a mouse model of *Escherichia coli* resulted in IL-1β–driven lethal immunopathology, which could not be tolerated by the host^34^. Inhibiting inflammasome signaling and pyroptosis by caspase-1 specific inhibitors or caspase-1 and NLRC4 knockout also significantly alleviated tissue damage, decreased bacterial burden, and increased the survival rate of infected mice^32^,^33^,^34^,^35^.

Many studies have proposed that pyroptosis may benefit the host during infection but may be detrimental during overwhelming infection or sepsis^12^,^13^. At the early onset of infection, bacteria hide in macrophages and replicate themselves until bacterial load is sufficient to subvert immune defense. Under such condition, pyroptosis is beneficial to the host because bacteria will be released from macrophages and subsequently killed by neutrophils. However, during overwhelming infection, inflammasome-induced pyroptosis releases a large number of pathogens from macrophages for dissemination throughout the host. The recruited neutrophils will fail to kill all bacteria and effectively control infection. Therefore, repeated rounds of pyroptosis can lead to severe inflammatory changes and significant damage to host tissues^36^.

Low bacterial quantities or localized infections induce inflammasome signaling and pyroptosis, and positively promote bacterial clearance^11^,^28^,^29^,^30^. High bacterial quantities or severe sepsis cause lethal inflammasome activation and pyroptosis^31^,^32^,^34^,^37^,^38^. In the present study, we performed intraperitoneal injection of 10^8^ CFUs of *S. Typhimurium* into mice. Such a high bacterial load could lead to severe and overwhelming sepsis-like infection. QseC blockade significantly suppressed flagellar gene expression in *S. Typhimurium* and subsequently inhibited caspase-1 activation, IL-1β release, and macrophage pyroptosis (Fig. 5), promoted the antimicrobial activity of macrophages to eliminate intracellular bacteria (Fig. 6), and alleviated inflammatory damage of infected tissues (Fig. 2).

The defensive function of inflammasome activation and pyroptosis as well as the role of IL-1β in the clearance of microbial infections are controversial. Pyroptosis is a caspase-1-dependent form of programmed cell death. Caspase-1 has a critical function in the cleavage of pro-IL-1β and in triggering inflammatory response^39^. IL-1β may induce the upregulation of adhesion molecules in the endothelium to mediate neutrophil recruitment and clear bacteria^40^. However, IL-1β is one of the major proinflammatory cytokines in sepsis^41^,^42^. While a low amount of these cytokines is critical for cell-mediated bacterial killing, excessive production of cytokines can lead to severe immunopathology^43^,^44^.

In summary, over-activation of inflammasomes can induce excessive macrophage pyroptosis and severe tissue damage. Our results demonstrated that QseC blockade significantly inhibited the pyroptosis of infected macrophages and improved the clearance of *S. Typhimurium* from infected macrophages.

## Additional Information

**How to cite this article**: Li, Z. *et al.* Pyroptosis of *Salmonella Typhimurium*-infected macrophages was suppressed and elimination of intracellular bacteria from macrophages was promoted by blocking QseC. *Sci. Rep.* **6**, 37447; doi: 10.1038/srep37447 (2016).

**Publisher’s note:** Springer Nature remains neutral with regard to jurisdictional claims in published maps and institutional affiliations.

## Supplementary Material

Supplementary Information

## Figures and Tables

**Table 1:** primers and plasmids used for *qseC* mutation in this study.

Primer		Sequence		
*qseC* up	Forward	5′-CACAGTGCCATAACGGCAACG-3′		
	Reverse	5′-GAAGCAGCTCCAGCCTACACTCATGCGTCACCCAGGGTGT-3′		
*qseC* down	Forward	5′-CTAAGGAGGATATTCATATGCAGAGACTTTTGCCAAAAACGC-3′		
	Reverse	5′-GAGGACGGCCTGACGGTGATGT-3′		
*cm*	Forward	5′-ACACCCTGGGTGACGCATGAGTGTAGGCTGGAGCTGCTTC-3′		
	Reverse	5′-GCGTTTTTGGCAAAAGTCTCTGCATATGAATATCCTCCTTAG-3′		
**Plasmids**		**Description**		
pKD3		pANTSγ derivative containing FRT-flanked chloramphenicol resistance		
pKD46		A red recombinase expression plasmid		
pCP20		An ampicillin and CmR plasmid that shows temperature-sensitive replication and thermal induction of FLP synthesis		

**Table 2:** Primers for real-time PCR used in this study.

Gene		Primer sequence
*flhDC*	Forward	5′-GTCAAACCGGAAATGACAAACTAA-3′
	Reverse	5′-ACCCTGCCGCAGATGGT-3′
*sifA*	Forward	5′-GTTGTCTAATGGAACCGATAATATCG-3′
	Reverse	5′-CTACCCCCTCCCTTCGACAT-3′
*sopB*	Forward	5′-CGGGTACCGCGTCAATTTC-3′
	Reverse	5′-TGGCGGCGAACCCTATAAA-3′
*rpoA*	Forward	5′-GCGCTCATCTTCTTCCGAAT-3′
	Reverse	5′-CGCGGTCGTGGTTATGTG-3′
