# Supplementary material for: Pyroptosis of Salmonella Typhimurium-infected macrophages was suppressed and elimination of intracellular bacteria from macrophages was promoted by blocking QseC
Source: Sci Rep. 2016 Nov 17;6:37447. doi: 10.1038/srep37447 (PMC5112599; doi:10.1038/srep37447)
Supplement: Supplementary Information [file srep37447-s1.doc]

Pyroptosis of *Salmonella Typhimurium*-infected macrophages was suppressed and elimination of intracellular bacteria from macrophages was promoted by blocking QseC

Zhi Li ^a,1^, Qing Zheng^b,1^, Xiaoyan Xue^a,1^, Xin Shi^a^, Ying Zhou^a^, Fei Da^a^, Di Qu^a^, Zheng Hou^a,*^ and Xiaoxing Luo^a,*^

^a^Department of Pharmacology, School of Pharmacy, Fourth Military Medical University, Xi'an 710032, PR China

^b^Center of Clinical Laboratory Medicine of PLA, Xijing Hospital, Fourth Military Medical University, Xi'an 710032, PR China.

*Corresponding author: Zheng Hou & Xiaoxing Luo

Tel/fax: +86 29 84774591.

E-mail address: hzh_0001@163.com (Z. Hou), xxluo3@fmmu.edu.cn (X.x. Luo)

^1^These authors contributed equally to this work.

**Supplementary Figure S1**


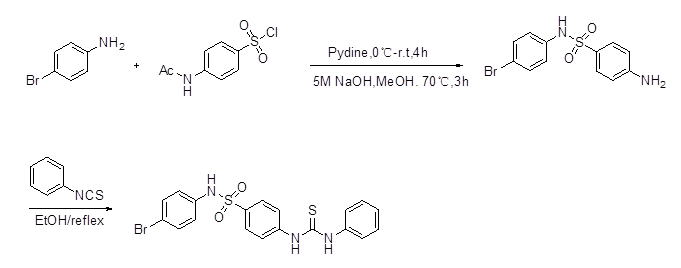


Fig. S1 The synthetic route and chemical structure of Br-LED209.
